# Successful Endoscopic Removal of a Clip‐stone Complex Following Laparoscopic Cholecystectomy: A Case Report

**DOI:** 10.1002/deo2.70321

**Published:** 2026-04-11

**Authors:** Takahiro Yamada, Masao Toki, Takuya Ishida, Hidenori Shibuta, Tadaaki Ogimoto, Sho Kawamoto, Kazushige Ochiai, Jun Miyoshi, Tadakazu Hisamatsu

**Affiliations:** ^1^ Department of Gastroenterology and Hepatology Kyorin University School of Medicine Tokyo Japan

**Keywords:** choledocholithiasis, clip migration, clip‐stone complex, endoscopic retrograde cholangiopancreatography, postcholecystectomy

## Abstract

An 84‐year‐old woman with a history of laparoscopic cholecystectomy for gallbladder stone disease 6 years earlier presented to our outpatient clinic. She was referred to gastroenterology for a thorough examination after blood tests showed liver dysfunction. An abdominal non‐contrast computed tomography scan showed a hyperdensity area in the common bile duct with a suspected metallic component, and we diagnosed a clip‐stone complex caused by a migrated clip. Laparoscopic cholecystectomy has become the standard of care for cholecystolithiasis, and as the number of operations increases, it is important to keep post‐cholecystectomy clip migration in mind as a late postoperative complication. We decided to perform endoscopic retrograde cholangiography to remove the complex stones, and performed a balloon catheter stone removal procedure with endoscopic papillary large balloon dilation (EPLBD). There have been reports of emergency surgery due to stone interference between the clip and catheter when a basket catheter was used to remove stones. We argue that balloon catheter stone removal in combination with EPLBD should be considered for clip‐stone complexes.

## 1 Introduction

Laparoscopic cholecystectomy has become the standard surgical procedure for treating choledocholithiasis. However, with advancements and developments in the procedure, including laparoscopic common bile duct exploration and increased use of surgical ligature clips, the incidence of post‐cholecystectomy clip migration (PCCM) has been increasing. We report our experience with a case of a complex stone formed with a surgical ligature clip as a nucleus, which was successfully removed endoscopically.

## 2 Case Report

**FIGURE 1:**
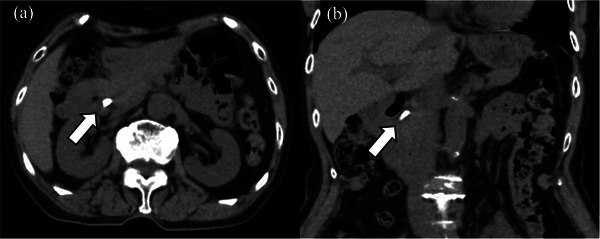
Non‐contrast computed tomography (CT) image((a) axial, (b) coronal) of the abdomen showed a hyperdense area (3137 Hounsfield units) in the distal bile duct.

**FIGURE 2:**
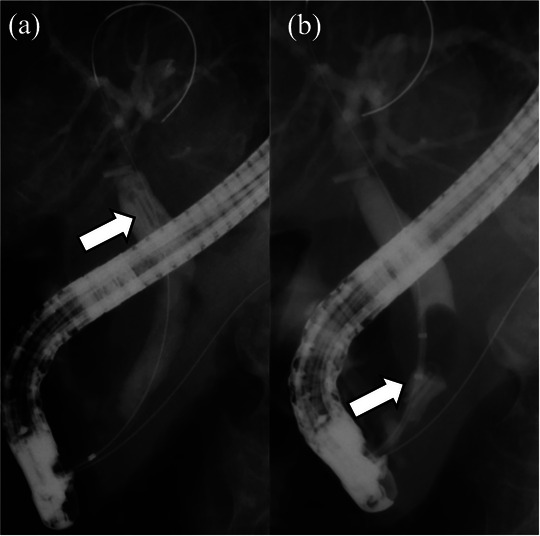
(a) Cholangiography showed a 10 × 4 mm defect (arrow) in the common bile duct. Metal was visualized within the defect. (b) A balloon catheter was used to remove the clip‐stone complex (arrow).

**FIGURE 3:**
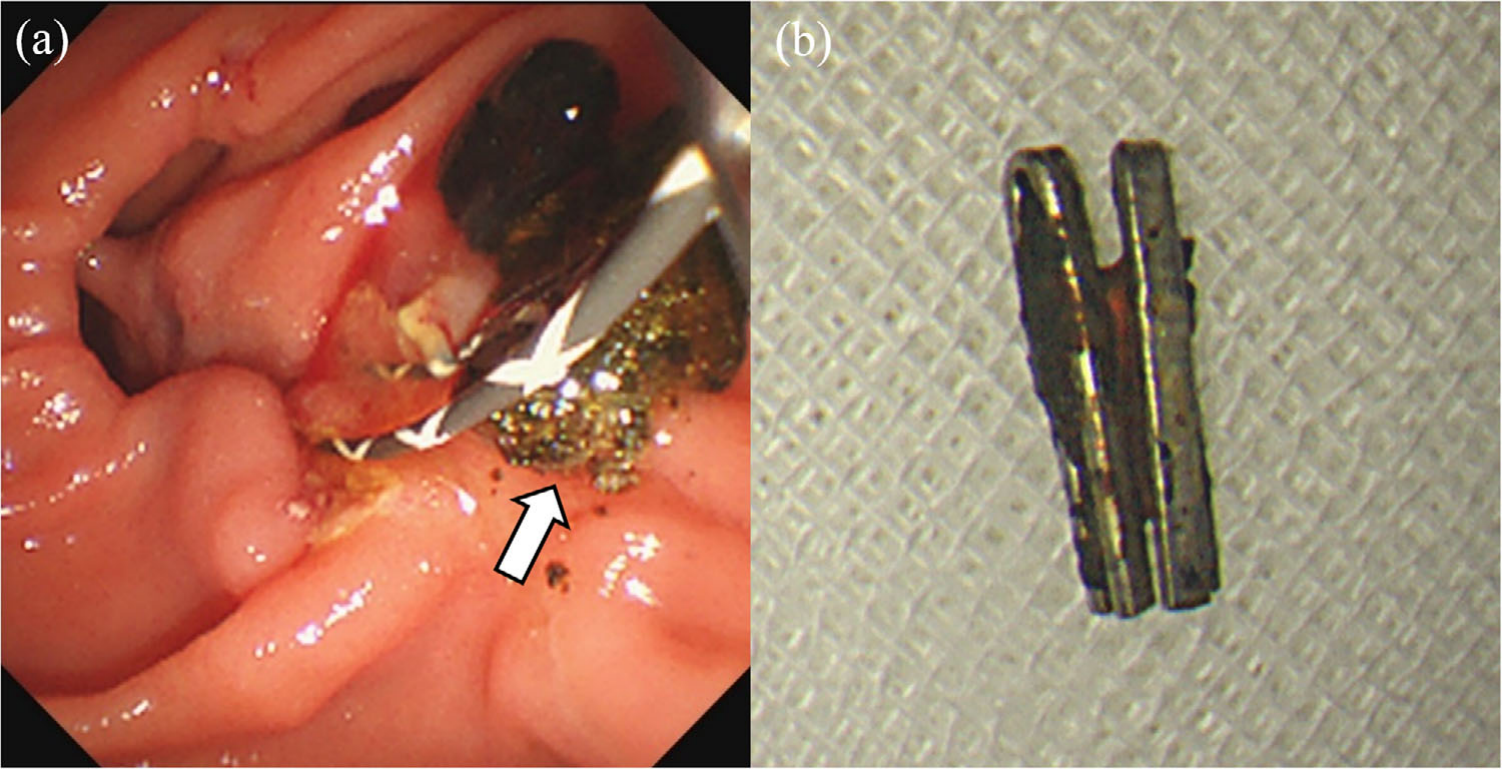
(a) An endoscopic image shows the clip‐stone complex being removed. (b) Photograph of the retrieved clip‐stone complex after removal.

An 84‐year‐old woman with a history of laparoscopic cholecystectomy for cholelithiasis 6 years prior and anti‐neutrophil cytoplasmic antibody‐related vasculitis was referred for evaluation of elevated transaminase concentrations. She had normal vital signs, no abdominal symptoms, and no findings indicative of jaundice. Laboratory data showed mild anemia, an elevated inflammatory response, elevated liver and biliary enzyme concentrations, but a normal serum bilirubin concentration. Non‐contrast computed tomography (CT) of the abdomen showed a clip‐shaped hyperdense area (>3000 Hounsfield units) in the distal bile duct (FIGURE 1). Endoscopic retrograde cholangiography showed a 10 × 4 mm defect containing metal within the common bile duct, and a migrated clip‐stone complex was diagnosed (FIGURE 2a). Endoscopic sphincterotomy was performed, followed by endoscopic papillary large‐balloon dilation (EPLBD). A balloon catheter was then used to safely remove the clip‐stone complex completely (FIGURE 2b). A metal clip was identified within the removed stone while performing the endoscopic procedure (FIGURE 3a). The clip was grasped using forceps and retrieved along with the endoscope (FIGURE 3b). Endoscopic retrograde cholangiography images (FIGURE 2b) revealed a clip in the hepatic hilum region; however, this clip was determined to be located outside the bile duct lumen, and we opted for observation. The patient experienced no postoperative complications and was discharged.

## 3 Discussion

The first case of PCCM into the common bile duct was reported by Onghena et al. in 1978 [1]. Laparoscopic cholecystectomy is a standard procedure performed over 100,000 times annually in Japan, and carries risks of serious postoperative complications such as bleeding, bile duct injury, and multiple organ failure. Although several cases of PCCM have been reported, its incidence remains low compared to the annual number of laparoscopic cholecystectomies performed. Koga et al. also described PCCM as a rare complication [2]. PCCM can cause acute pancreatitis, common bile duct stones, and clip‐stone complex formation. The median time from cholecystectomy to PCCM occurrence in Japan is 3 years [3]; however, occurrence as late as 16 years has been reported [4]. We should keep PCCM in mind PCCM as a potential late complication of laparoscopic cholecystectomy.

Although various factors have been identified, a definitive mechanism by which clips migrate into the common bile duct has not yet been identified. Theories proposed include compression by surrounding organs [5], and the clip is pulled into the common bile duct during the resorption process of the biloma [6], microabscess formation around the clip owing to bile duct injury or local inflammation leading to erosion into the bile duct wall [7], and the sharp edge of the clip makes contact with the bile duct wall and gradually erodes it with minute vibrations [8]. Individual conditions, environments, and the number of clips may also influence the occurrence of PCCM.

Suzuki et al. investigated the origins of migrated clips, finding that clips used to occlude the cystic duct were the most common [3]. However, clips used for cystic artery ligation, hepatic hemostasis, and reinforcement of C‐tubes were also identified. Therefore, migrated clips are not necessarily limited to those placed around the Calot triangle. During a laparoscopic cholecystectomy, two clips are typically placed on the proximal side of the cystic duct, and one or two clips are placed on the cystic artery to perform gallbladder resection. Suzuki et al. reported an increased risk of PCCM in cases where five or more metal clips were used, suggesting that minimizing the number of foreign bodies is an effective preventive measure against PCCM [3]. It should be noted that the use of absorbable sutures and clips cannot completely prevent this condition, as there are reports of complex stones with silk thread cores, not limited to metal clips. In this case, two clips were applied to both the cystic duct and cystic artery, resulting in a total of four clips. Although detailed operative information, such as the clip type and the exact distance from the common bile duct, was not available, CT images suggested that metal clips had been used.

In this case, we diagnosed PCCM based on abdominal computed tomography. In patients with elevated liver and biliary enzyme concentrations after laparoscopic cholecystectomy, it is important to evaluate imaging findings with PCCM in mind. Ali et al. reported no significant difference in success rates between surgical and endoscopic treatments for PCCM [9]. We elected to perform endoscopic clip‐stone complex removal, similar to how we would manage common bile duct stones. Because a previous report of endoscopic stone removal using a basket catheter resulted in failure owing to interference between the clip and catheter, and surgical removal was required [10], we performed EPLBD and used a balloon catheter for removal. Balloon catheter‐based stone removal in combination with EPLBD should be considered as a treatment modality for clip‐stone complexes.

## Author Contributions

**Takahiro Yamada**: conceptualization, methodology, data curation, investigation, visualization, project administration, writing – original draft, and writing – review & editing. **Masao Toki**: conceptualization, methodology, data curation, investigation, supervision, visualization, project administration, writing – original draft, and writing – review & editing. **Takuya Ishida**: writing – review & editing. **Hidenori Shibuta**: writing – review & editing. **Tadaaki Ogimoto**: writing – review & editing. **Sho Kawamoto**: writing – original draft and writing – review & editing. **Kazushige Ochiai**: writing – original draft and writing – review & editing. **Jun Miyoshi**: writing – review & editing. **Tadakazu Hisamatsu**: conceptualization, methodology, supervision, project administration, writing – original draft, and writing – review & editing.

## Funding

No funding was received for this manuscript.

## Conflicts of Interest

Tadakazu Hisamatsu has received grant support from Mitsubishi Tanabe Pharma Corporation and EA Pharma Co., Ltd., AbbVie GK, JIMRO Co., Ltd., Zeria Pharmaceutical Co., Ltd., Nippon Kayaku Co., Ltd., Takeda Pharmaceutical Co., Ltd., Pfizer Inc., Boston Scientific Corporation, Mochida, and Meiji HD. Tadakazu Hisamatsu has also received consulting and lecture fees from EA Pharma Co., Ltd., AbbVie GK, Janssen Pharmaceutical K.K., Pfizer Inc., Mitsubishi Tanabe Pharma Corporation, JIMRO Co., Mochida Pharmaceutical Co., Ltd., Bristol Myers Squibb Co., Eli Lilly and Company, Gilead Sciences Inc., Bristol Myers Squibb Co., Abivax, Chugai, MSD, Nippon Kayaku Co., Ltd., and Kyorin Pharmaceutical Co., Ltd.

The other authors declare no conflicts of interest.
